# Partial Discharge Recognition with a Multi-Resolution Convolutional Neural Network

**DOI:** 10.3390/s18103512

**Published:** 2018-10-18

**Authors:** Gaoyang Li, Xiaohua Wang, Xi Li, Aijun Yang, Mingzhe Rong

**Affiliations:** 1 State Key Laboratory of Electrical Insulation and Power Equipment, School of Electrical Engineering, Xi’an Jiaotong University, Xi’an 710049, China; ligaoyang@stu.xjtu.edu.cn (G.L.); yangaijun@mail.xjtu.edu.cn (A.Y.); 2 The Global Energy Interconnection Development and Cooperation Organization (GEIDCO), Beijing 100031, China; xi-li@geidco.org

**Keywords:** partial discharge, ultra-high-frequency signals, multi-resolution analysis, convolutional neural network

## Abstract

Partial discharge (PD) is not only an important symptom for monitoring the imperfections in the insulation system of a gas-insulated switchgear (GIS), but also the factor that accelerates the degradation. At present, monitoring ultra-high-frequency (UHF) signals induced by PDs is regarded as one of the most effective approaches for assessing the insulation severity and classifying the PDs. Therefore, in this paper, a deep learning-based PD classification algorithm is proposed and realized with a multi-column convolutional neural network (CNN) that incorporates UHF spectra of multiple resolutions. First, three subnetworks, as characterized by their specified designed temporal filters, frequency filters, and texture filters, are organized and then intergraded by a fully-connected neural network. Then, a long short-term memory (LSTM) network is utilized for fusing the embedded multi-sensor information. Furthermore, to alleviate the risk of overfitting, a transfer learning approach inspired by manifold learning is also present for model training. To demonstrate, 13 modes of defects considering both the defect types and their relative positions were well designed for a simulated GIS tank. A detailed analysis of the performance reveals the clear superiority of the proposed method, compared to18 typical baselines. Several advanced visualization techniques are also implemented to explore the possible qualitative interpretations of the learned features. Finally, a unified framework based on matrix projection is discussed to provide a possible explanation for the effectiveness of the architecture.

## 1. Introduction

Gas-insulated switchgears (GISs) are widely used as the major control and protection equipment in medium to ultra-high voltage substations, due to their superior compactness, high reliability, strong dielectric strength, and maintenance-free properties. However, unavoidable imperfections and deterioration in the insulation system pose serious threats to the reliability and safety of the GIS and whole power grids [1,2,3]. The occurrence of partial discharges (PDs) is not only one of the main characteristics that can effectively reflect the inner dielectric flaws, but is also the cause of the accelerated degradation [4,5]. Therefore, it is imperative to assess the potential correlation between the PD patterns and the defect types, so that corresponding maintenance activities can be taken before a complete breakdown.

The conventional monitoring methods of PD could be summarized into three main categories: chemical, electrical, and acoustic [6]. Among these, ultra-high-frequency (UHF) signals are becoming increasingly important due to their high sensitivity, better immunity against interference, and the ability for real-time monitoring [7,8]. Considering the stochastic nature of PDs, machine learning has long been the mainstream method for the diagnosis of UHF signals. The existing literature covers a wide range of techniques, including Support Vector Machines (SVM) [9], Neural Networks (NN) [10,11,12], cluster analysis [13], fuzzy logic [14] and so on. A more detailed taxonomy of NNs could give rise to methodologies such as the self-organizing map (SOM) [10], Ensemble neural networks (ENN) [11], and Probabilistic Neural Networks (PNN) [12]. Compared with classifiers, feature extraction plays a more dominant role in the success of PD recognition due to the high dimensionality of the PD data. A gamut of techniques have been applied to both the time-resolved partial discharge (TRPD) and phase-resolved partial discharge (PRPD) patterns [6], of which the statistical parameters [15], the time-power ratio map [16], fractal features [17], Fourier transform [18], wavelet analysis [12], Hilbert-Huang transform (HHT) [19], and S-transform [20] are a few. In addition, some advanced signal processing techniques such as compressed sensing [21] and principal component analysis (PCA) [22] are also utilized. A summary of these implementations is shown in Table 1.

Although the cooperation of features and algorithms indicate a considerable degree of success, it is pertinent to note that most of them suffer from a fundamental limitation: the carefully selective feature extraction process usually calls for much human expertise and hence, it is subject to human error. The significant randomness of the electromagnetic waves caused by propagation and reflection makes the process even more intricate [23]. Besides, even with all these efforts, the features designed for a particular task are not guaranteed to be the optimal choices for the other application scenarios and datasets. Therefore, learning features directly from the dataset itself may be a better choice.

Among all of the analysis methods, time–frequency (TF) analysis is especially applicable, due to its invertibility, and its ability to provide both time and frequency resolutions [24]. From the perspective of manifold learning, the intrinsic advantage of the TF method is to obtain continuity and compactness in a high-dimensional space at the cost of dimensionality. However, the high dimensionality poses a substantial impediment for the traditional machine learning models. The matrix compression algorithms for dimensionality reduction such as the two-dimensional PCA (2DPCA) [25] and Non-negative matrix factorization (NMF) [26] inevitably share similar obstacles with the other feature engineering-based methodologies, such as adaptation and generalization.

Fortunately, the recent advances in deep learning make it possible to extract high-level features automatically from high-dimensional sensory data, and they demonstrate dramatic success in various areas such as natural language processing, image classification, and auto-driving, of which the Convolutional Neural Network (CNN) has been designed for vision-related tasks [27,28]. Therefore, the combination of the CNN and TF maps becomes a potentially promising option for the PD signal recognition tasks. Nevertheless, although it sounds transparent, directly applying CNN for TF maps is not a good idea, due to the inherently non-local and serial characteristics of TF maps. In our preliminary work [29], we proposed a CNN of frequency matching to obtain the semantic meaning. In this paper, we further improve the framework into multiple columns to realize multi-resolution diagnosis, thus promoting the diagnostic accuracy to a new level. The major contributions are summarized as follows.

1. We propose a deep multi-column architecture that incorporates multi-resolution information for the recognition of UHF signals with specified temporal filters, frequency filters, and texture filters. Multi-sensor fusion based on LSTM is also utilized to further improve the diagnostic accuracy.
2. To alleviate the risk of overfitting induced by the expanded structure, a spectral embedding technique based on manifold learning is utilized for the pretraining. We demonstrate that the transfer learning-based training process is able to achieve remarkable performance with limited data.
3. A unified matrix projection-based framework is proposed to enhance the interpretability of the proposed model.

The rest of this paper is organized as follows. Some related theories are introduced first in Section 2. Section 3 provides the details of both the model architectures and the transfer learning process. The platform presented in Section 4 provides the necessary dataset for verifying the proposed method, and Section 5 describes the recognition results. Based on the experimental results, Section 6 discusses the effectiveness of the proposed model. Finally, Section 7 concludes the paper.

## 2. Related Work

In deep learning, the most straightforward way to improve the model performance has shifted from feature engineering to network architecture designing. ResNet [30] proposed by Microsoft is a good example, which introduces an extra column with residual mapping, and has state-of-the-art performance. Although the early implementation of the multi-column neural network only aimed at accelerating the training process with multiple Graphics Processing Units (GPUs) [28], it has been realized that the several columns can become specified experts for different features. For example, the Inception model [31] is a successful multi-column deep neural network (DNN) to approximate the optimal sparse structure of the input with different convolutional kernels. The Xception model [32] goes even further by decoupling both the cross-channel and spatial correlations to obtain a multi-branch structure. In addition, Saining exposed a new dimension called cardinality to standardize the multi-branch structure [33]. Ling attempted to train a multi-column neural network with spectral clustering to embed the hierarchical manifolds into a compact vector [34].

Although sharing the similar structure of multiple columns, the multi-resolution theories focus more on the structures of different scales. Sander attempted to learn music features by extracting the mel-spectrograms with different windows [35]. Michael utilized CNN to predict future images from different resolutions and concluded that a lower resolution was able to capture the entire context, while a higher resolution recovered more details [36]. The similar idea was also shared by MMDenseNet [37], which enables the neural network to model both the fine-grained structures and the long contexts efficiently. In the Deep Belief Network, the multi-resolution structures also showed their superiority in image-related tasks [38]. Furthermore, the idea of multiple resolutions has long been integrated into the underlying design of neural networks. The pooling layers and the variants, such as subsequent spatial pyramid pooling all attempt to obtain the larger space by reducing the resolution of the intermediary layers [39].

## 3. Proposed Method

### 3.1. Gabor Representations and Multi-Resolution Analysis

Fourier transform is a powerful tool to provide the frequency distributions of complex signals. However, we must simply remark that the one-dimensional solutions are delocalized in time and not comprehensive enough. The bidimensional functions that provide both the frequency and time localizations are preferred. To introduce the time resolution, a time-localized window is utilized to suppress the signal outside the neighborhood, which is the well-known short-time Fourier transform (STFT) [24]:

(1) $$F_{x}\left(t,v;h\right)=\int_{-\infty }^{+\infty }x\left(u\right)h^{*}\left(u-t\right)exp\left[-j2\pi vu\right]du$$

where x(u) is the original signal, and h is the analysis window. t and v represent the time and frequency, respectively. In practical applications, the discrete STFT is preferred, which is given by:

(2) $$F_{x}\left[n,m;h\right]=F_{x}\left(nt_{0},mv_{0};h\right)=\int_{-\infty }^{+\infty }x\left(u\right)h^{*}\left(u-nt_{0}\right)exp\left[-j2\pi mv_{0}u\right]du$$

where m,n∈Z. $t_{0}$ and $v_{0}$ are the time and frequency steps. Specifically, the STFT with Gaussian windows is able to theoretically maximize the concentration in the TF plane by approaching the lower bound of the Heisenberg–Gabor inequality [24]:

(3) T×B≥1

**Figure 1:**
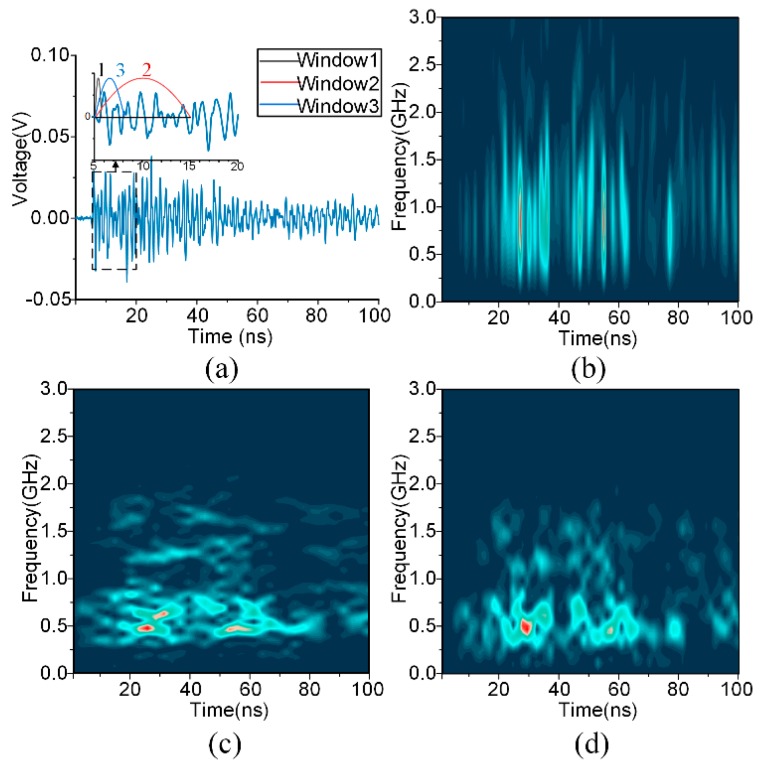
Gabor representations of different time and frequency resolutions: (**a**) windows of different lengths; (**b**) spectrogram of higher time resolution; (**c**) spectrogram of higher frequency resolution; (**d**) spectrum of medium resolution.

Here, T is the time duration, and B is the normalized bandwidth. The STFT of this particular case is also called the Gabor transform. The most important inference of the Heisenberg-Gabor inequality is the trade-off between time and frequency resolutions in accordance with the width of the analysis window. An illustrative example is shown in Figure 1.

As shown in Figure 1, the long window gives rise to harmonic information, while the pulses emerge from the short time window. In this study, the spectrograms of different resolutions are all employed for the diagnosis of the UHF signals. It should be noted that the concept of multiple resolutions is different from the wavelet or S-transform, which still generate only a single resolution for each frequency band.

**Figure 2:**
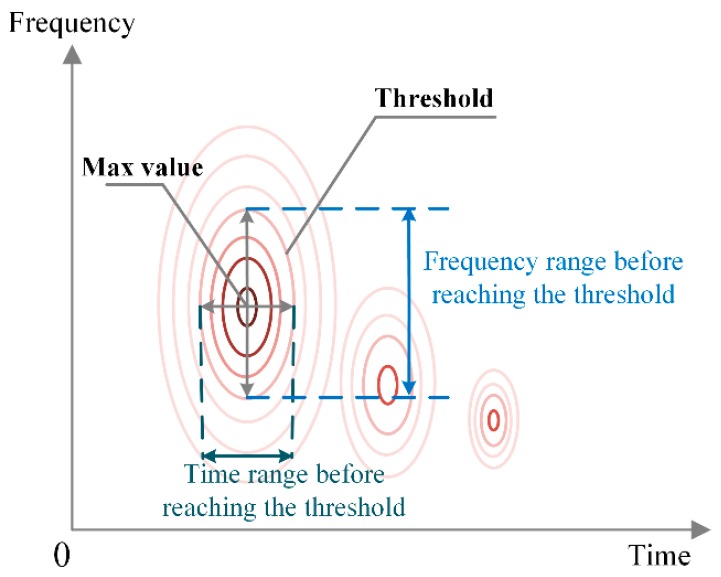
Schematic diagram of the time range and frequency range before reaching the threshold.

Furthermore, a semiquantitative method is proposed as an assistance criteria for choosing the proper analysis windows, considering the morphology characteristics of the STFT spectrums. As indicated by Figure 2, centering on the max value, the time and frequency ranges before reaching a certain threshold are calculated, which can be regarded as a rough description of the peak shape. Intuitively, the greater the time range is, the less the time resolution is retained, while the frequency isolation is more obvious. The frequency range varies in an opposite way. The proper window can be selected based on the trade-off between the time and frequency resolutions, depending on their priorities.

### 3.2. Convolutional Neural Network and the Design of Convolutional Kernels

In recent years, CNN and its derivatives have been regarded as the state-of-the-art choices for computer vision tasks. The key ideas in CNN include the concepts of local receptive fields, shared weights, and spatial subsampling. Specifically, in each convolutional layer, the feature maps are first convolved with several kernels. After the biases, activation function and sometimes, the pooling operation, the outputs are then fed into the next convolutional layer, which can be summarized as:

(4) $$x_{j}^{l}=f\left(\underset{i\in M_{j}}{\sum }x_{i}^{l-1}*k_{ij}^{l}+b_{j}^{l}\right)$$

where ∗ donates the convolutional operator. $x_{j}^{l-1}$ and $x_{i}^{l}$ are the input and output of the *l*th layer, respectively, and b is the bias. $M_{j}$ is a selection of the feature maps, and $k_{ij}^{l}$ is the element of the kernel matrix.

The activation function f is chosen as the Relu function for the convenience of the gradient calculation. A simple parameter analysis can be given as follows. Assuming that the volume of input is W1×H1×D1, after applying convolutions of k filters (without zero padding), the new volume of W2×H2×D2 is:

(5) $$W2=\frac{\left(W1-F1\right)}{S1}+1$$

(6) $$H2=\frac{\left(H1-F2\right)}{S2}+1$$

(7) D2=k

where F1 and F2 are the width and height of the filter cores, and S1 and S2 are the strides. One special characteristic of CNN is the shift invariance. However, although dramatically useful in image recognition, the invariance features pose an impediment for the recognition of Gabor representations that own a clear physical meaning. Any shift of a generally rectangular filter may distort the original TF distributions and confuse the CNN. To alleviate the restriction, three kinds of convolutional kernels are designed in accordance with the spectrograms of different resolutions, namely frequency filters, temporal filters, and texture filters.

**Figure 3:**
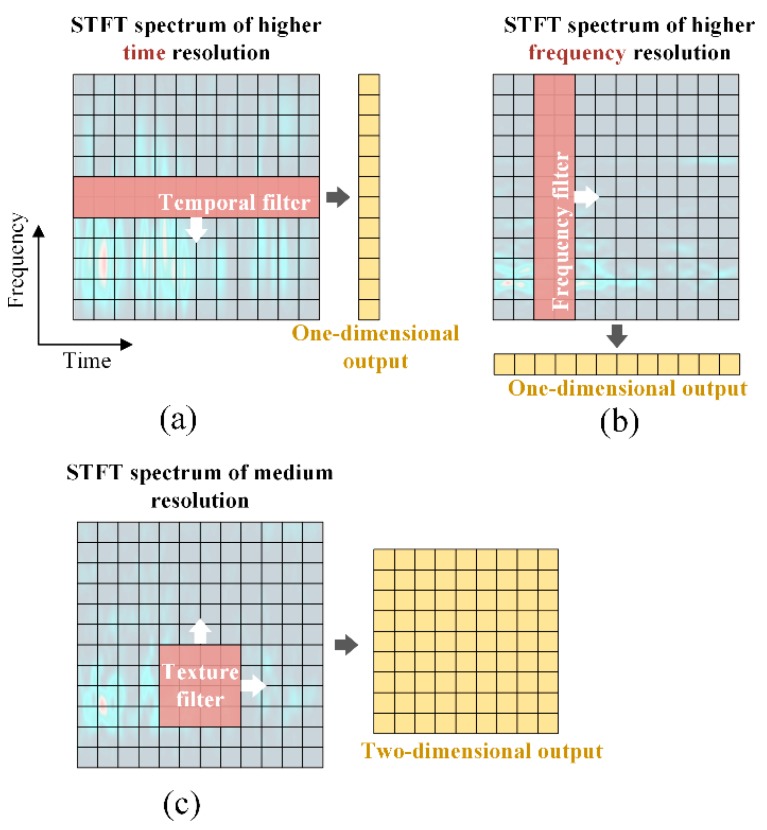
Schematic diagram of the different convolutional filters and their corresponding outputs: (**a**) temporal filter; (**b**) frequency filter; (**c**) texture filter.

As shown by the red rectangles in Figure 3, the temporal filters are designed for maps of higher time resolutions, and they share the same widths with the maps, while the frequency filters are suitable for higher frequency resolutions, and they are as high as the spectrograms. The texture filters are of medium size as supplements.

The designing principles of the special convolutional filters may be explained qualitatively with orthogonality, since the convolutional operation is basically a template matching process. The first step is to make the filter cover either a complete frequency axis or a whole time axis, thereby allowing for perfect frequency or temporal matching. Second, by assuming that the filter is also a kind of special signal, it is assumed that orthogonality exists between the unfolding direction of the filters and the spectrograms, to maximize the diversity in a filter as much as possible. Following this idea, the frequency filters are applied to the Gabor maps of higher frequency resolutions, while the temporal filters are applied to the higher time resolutions. Another advantage of the frequency and temporal filters refers to Equations (5) and (6). The outputs of the frequency and temporal filters are only one-dimensional, which dramatically decreases the model’s size.

### 3.3. Architecture of the Proposed Model

As has been mentioned before, TF maps of different resolutions can reveal different rhythms in the 2-dimensional space. It is hypothesized that it would be of great benefit to explicitly provide TF maps of different resolutions as inputs to a deep model. This combines the multiple views with deep learning’s ability to extract intrinsic properties from raw inputs, so that the discriminative features could be learned while maintaining explanatory factors with physical meaning.

**Figure 4:**
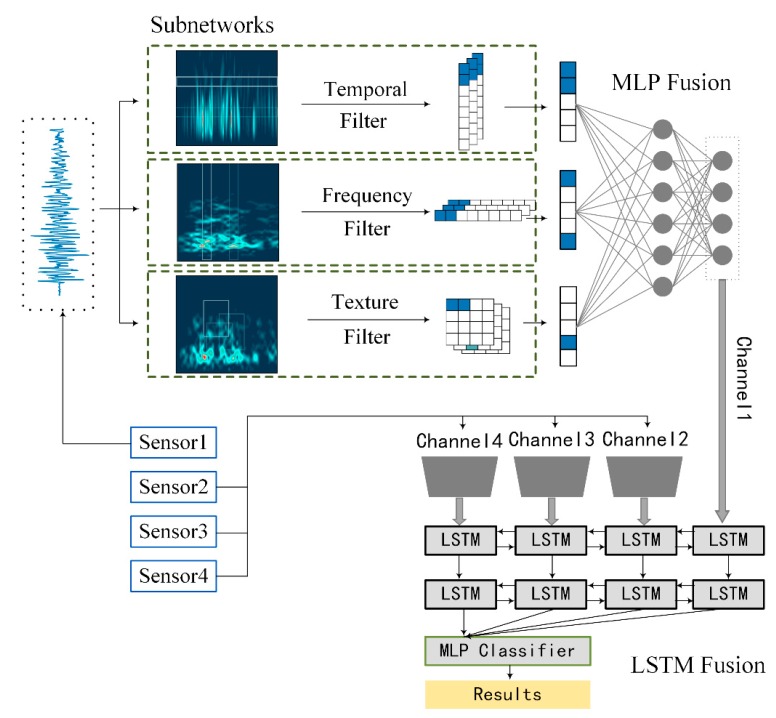
Structure of the multi-resolution CNN and the LSTM fusion.

Accordingly, the proposed multi-resolution network is summarized in Figure 4. It mainly contains three parts, namely, single-resolution embedding, multi-resolution fusion, and multi-sensor fusion. Here, the first part is realized by three subnetworks; the second part is a fully-connected neural network attached at the end of the subnetworks, and Long Short-Term Memory (LSTM) [40] is implemented for multi-sensor fusion.

1. Single-resolution embeddings with the subnetworks

Each branch of the subnetworks has its own micro-structure, which is basically a CNN with the convolutional operation. The main differences lie in the specified designed frequency filters, temporal filters, and texture filters. After the convolutional filter, an activation function, a pooling layer, and a dropout layer are arranged hierarchically as a basic building block, which repeats itself twice before connecting to a flattening layer. Finally, a fully-connected layer is attached at the end of each subnetwork to generate an embedding vector as the intermediate feature space.

2. Multi-resolution information fusion using the fully-connected neural network

The fully connected neural network is a normal multi-layer perceptron (MLP) containing two layers of 100 units and 50 units, respectively, that are attached after the concatenation of the three subnetworks. The model automatically chooses the best filters by assigning them higher weights, and the information of different resolutions is merged to offer comprehensive representations of compact forms.

3. Multi-sensor information fusion using the LSTM

The propagation and reflections of UHF signals in the GIS can significantly influence the characteristics of the signals, especially at the L corner for the inconsistency of the wave impedance [41]. Therefore, the characteristics of sensors of different positions can reveal different patterns. By fusing the information from different sensors, it is possible to obtain a more comprehensive understanding of the PD patterns.

**Figure 5:**
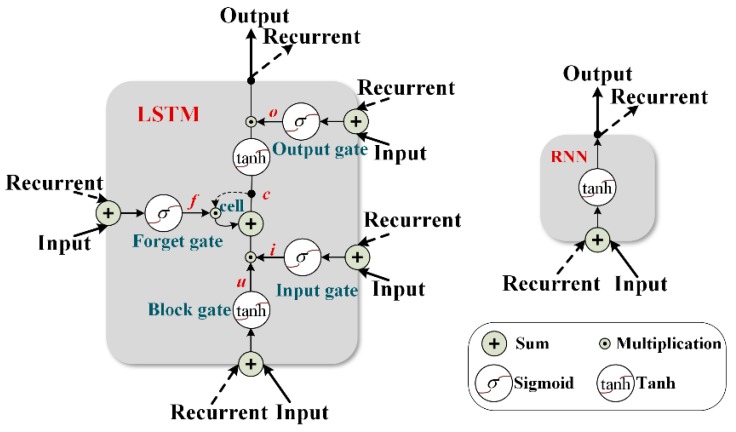
Architectures of LSTM and a general RNN.

Recurrent neural network (RNNs) [42] is the most popular choice for sequence mining tasks such as speech recognition, natural language translation, and video recognition. As shown in Figure 5, in each time step t, the hidden state $h_{t}$ is updated by a function of the last hidden state $h_{t-1}$ and the new input $x_{t}$:

(8) $$\;h_{t}=tanh\left(Wx_{t}+Uh_{t-1}+b\right)$$

where the hidden state $h_{t}$ is a d-dimensional vector, and b is the bias. W and U are the weights. Tanh is the activation function. LSTM is a branch of RNN, which introduces a memory cell to preserve the information for long sequences, and relieves the exploding or vanishing gradients problem in the original RNN [40]. The basic unit of LSTM can be explained as a collection of a block input $x_{t}$, a memory cell $c_{t}$, and a hidden state $h_{t}$, controlled by an input gate $i_{t}$, a forget gate $f_{t}$, and an output gate $o_{t}$ as shown in Figure 5, with the following relationships:

(9) $$i_{t}=\sigma \left(W^{\left(i\right)}x_{t}+U^{\left(i\right)}h_{t-1}+b^{i}\right)$$

(10) $$\;f_{t}=\sigma \left(W^{\left(f\right)}x_{t}+U^{\left(f\right)}h_{t-1}+b^{f}\right)\;$$

(11) $$\;o_{t}=\sigma \left(W^{\left(o\right)}x_{t}+U^{\left(o\right)}h_{t-1}+b^{o}\right)\;$$

(12) $$\;u_{t}=tanh\left(W^{\left(u\right)}x_{t}+U^{\left(u\right)}h_{t-1}+b^{u}\right)\;$$

(13) $$\;c_{t}=i_{t}⊙u_{t}+f_{t}⊙c_{t-1}\;$$

(14) $$\;h_{t}=o_{t}⊙tanh\left(c_{t}\right)\;$$

where *σ* is the sigmoid function and ⊙ donates the point multiplication. $W^{\left(i\right)}$, $W^{\left(f\right)}$, $W^{\left(o\right)}$, $W^{\left(u\right)}$, $U^{\left(i\right)}$, $U^{\left(f\right)}$, $U^{\left(o\right)}$, and $U^{\left(u\right)}$ are the weights. $b^{i}$, $b^{f}$, $b^{o}$, and $b^{u}$ are the biases. Intuitively, the input gate and the output gate control how much the old memories and new information are recombined as the new memory cell. The hidden state $h_{t}$ is a gated, partial view of the internal memory of the cell. By varying the gate values in different time steps, the LSTM can keep itself up-to-date while maintaining the most discriminable information acquired from different time steps. Finally, the features from different sensors are integrated for information fusion to give the final diagnosis results.

As the net structure is very complex, more sophisticated techniques are required to relieve the risk of overfitting. Details of the training process will be presented in the next part.

### 3.4. Model Training by Transfer Learning

Although the multi-resolution views and the multi-steam structures gain great potential for more comprehensive diagnosis, a larger number of parameters accompanies its bigger size, making the model more prone to overfitting. Thus, a transfer learning framework was proposed that classifies the training process into the three stages of single-resolution embedding, multi-resolution fusion, and multi-sensor fusion, in accordance with the network’s structure.

Transfer learning is a technique focusing on transferring knowledge gained from one problem to a different but related task [43]. The parameters of the target network are partly or all initialized from another network that is pretrained from the source task, while the nontransferred weights are randomly initialized. In most cases, transfer learning is done by weight transfer.

**Figure 6:**
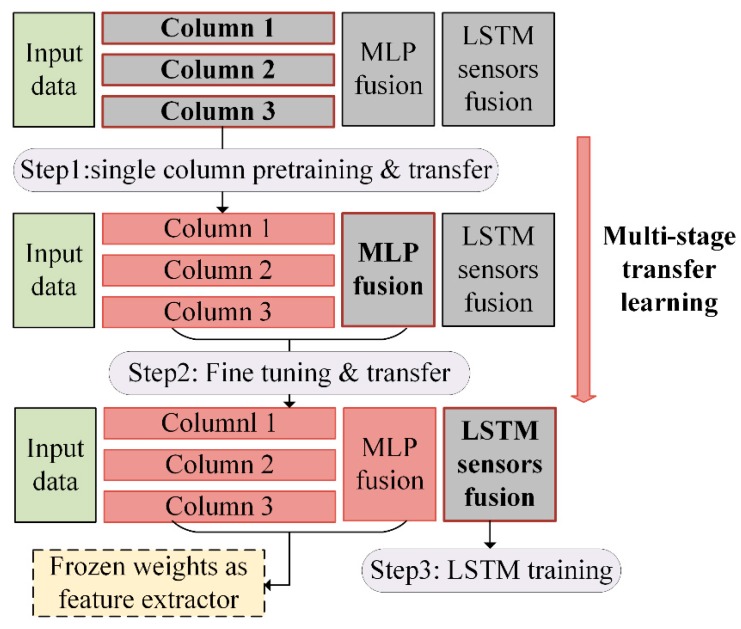
Three stages of the knowledge transfer process.

The usage of transfer learning was more flexible in this study, where the source and target problem use the same dataset. The key idea is relative independence among columns of different resolutions. The same dataset is much less prone to overfitting when it is applied to a smaller problem. Therefore, it is beneficial if the single-column is trained first independently. The knowledge is then transferred to the higher levels for more sophisticated targets. The details of the three learning stages are shown in Figure 6.

1. The single-column features extracted by each subnetwork are learned using manifold embedding.
2. The weights of the MLP attached at the end of the three subnetworks are randomly initialized, and the whole structure is fine-tuned.
3. The weights gained from step (2) are frozen as feature extractors. Only the weights of the LSTM are updated for multi-sensor fusion.

#### 3.4.1. Single Column Embedding with Manifold Learning

It is worth noting that the subnetwork that embeds the high-dimensional data into a fixed vector is similar to a general dimensionality reduction problem that maps the high dimensional points onto a low dimensional manifold to make the similar points adjacent. Although some general dimensionality reduction frameworks exist [44,45], the same key point is to preserve the distances of the original vertex pairs. Given the data $x_{1,\ldots ,}x_{U}$, the loss function parameterized by *α* and embedding function f is:

(15) $$Loss=\overset{U}{\underset{i,j}{\sum }}L\left(\|f\left(x_{i},\alpha \right)-f\left(x_{j},\alpha \right)\|-W_{ij}\right)\;$$

where $W_{ij}$ is the element of a pairwise distance matrix in a class-dependent or class-independent way. For example, Multidimensional scaling (MDS) [46] can be used in this pattern by defining $W_{ij}$ as the Euclidean distance. For the specified problem, the distances are defined in a class-dependent way as follows. $W_{ij}=0$ if i and j belong to the same class, and 1 otherwise.

**Figure 7:**
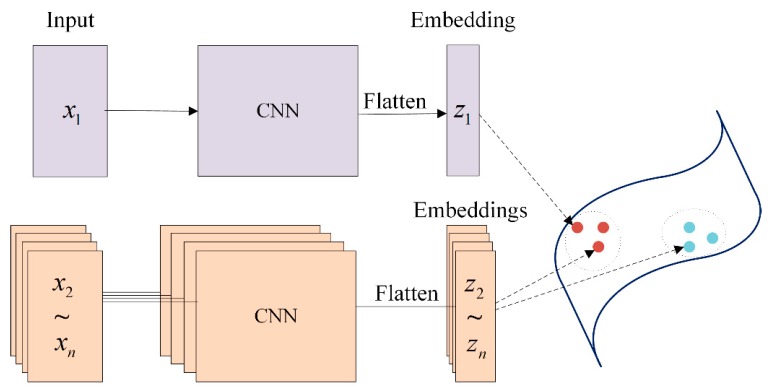
Siamese network for embedding the feature maps into a constant vector.

Furthermore, to make the loss function learnable and more suitable for the subnetworks, a Siamese network structure [47] is implemented to realize the pairwise affinity calculation. A Siamese network is a special neural network with two symmetric branches, as shown in Figure 7. Each time, only one subnetwork is fabricated into the Siamese structure for pretraining with the following loss function:

(16) $$\;Loss=\{\begin{array}{ll} \|f\left(x_{i},\alpha \right)-f\left(x_{j},\alpha \right){\|}^{2} & if\;W_{ij}=0\\ max\left(0,m-\|f\left(x_{i},\alpha \right)-f\left(x_{j},\alpha \right){\|}^{2}\right) & if\;W_{ij}=1 \end{array}$$

where m is the margin. The loss function is different from MDS in two ways. First, the distance is class-dependent. Second, the losses are chosen as the hinge losses for better generalization. After accomplishing the model construction, Adadelta optimizer is utilized for the weights update.

#### 3.4.2. Transfer Learning for Cascaded Training

For the other two steps, the weights of the MLP that were attached at the end of the three subnetworks were randomly initialized first, and then another layer with the same node number of the classes was connected to make a complete single-sensor diagnosis model. After fine-tuning with Adadelta, the weights gained from step (2) were frozen, and they only acted as feature extractors for the information fusion of different sensors, while the weights of the LSTM were made to be trainable. The convergence speeds of the two steps were likely to be much faster compared with the first step, due to the existing class-dependent knowledge. In addition, some advanced training techniques such as early stopping and heuristic learning rate adaption were also utilized.

## 4. PD Laboratory Setup

**Figure 8:**
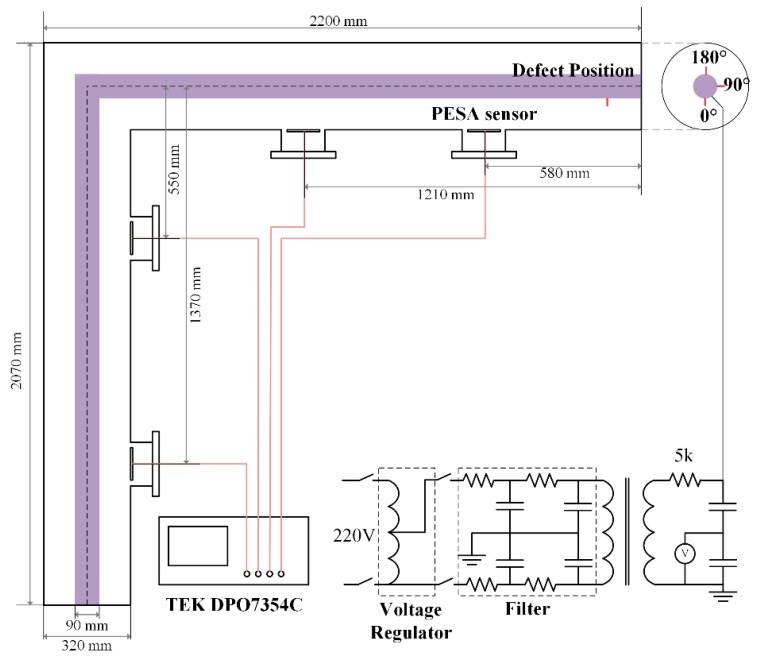
Schematic diagram of the GIS tank, PESA sensors, and the experimental circuit.

**Figure 9:**
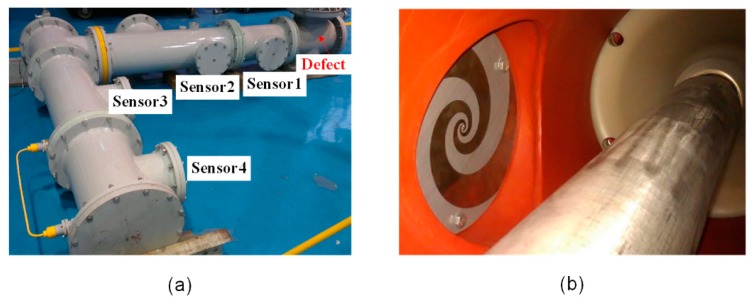
GIS experimental platform: (**a**) GIS tank and the positions of the UHF sensors; (**b**) the installation of the UHF sensors inside the tank.

For the purpose of the experimental study, a simulative L-shaped GIS tank with a coaxial cylindrical conductor was implemented to simulate the realistic equipment, as shown in Figure 8 and Figure 9a, with the central conductor diameter being 90 mm, and the enclosure tank diameter being 320 mm. The tank was filled with SF6 gas of 0.1 MPa to simulate the propagation medium.

Four planar equiangular spiral antennas (PESAs) with impedance transformers were installed inside the GIS chamber in the hand holes parallel to the axis of the GIS bus-bar, as shown in Figure 8 and Figure 9b. The PESA utilized in this study is an ultra-wideband antenna that can be installed internally in the GIS. Its outside and inside radii are 109 mm and 2 mm, respectively, with the substrate thickness being 1 mm. The other key parameters that can affect the performance of the PESA sensors include the relative dielectric constant, the spiral growth rate, and the rotation angle, which are chosen as 2.65, 0.364, and 3.5π, respectively. In practice, the most commonly quoted parameter to evaluate the performance of the antenna is the reflection coefficient *S*_11_, which represents how much power is reflected from the antenna. The range of the bandwidth that satisfies *S*_11_ < −10 dB was 0.8 GHz to 3.5 GHz from the lower limit to the higher limit for the PESAs. Besides, the *S*_11_ parameter ranged between −8 dB to −6 dB from 0.2 GHz and 0.7 GHz, and close to −10 dB in the 0.7 GHz to 0.8 GHz range, which satisfied the measurement requirements of the UHF signals. The detailed *S*_11_ curve can be found in our previous study [48]. The geometrical dimensions of the GIS tank and the relative distances between the PESAs are also illustrated in Figure 8.

High voltage was introduced by an insulating bushing connected with a non-PD testing transformer (Xinyuan Electric, Yangzhou, China, YDTW-30/150) with an amplitude of 0–150 kV, and a capacity of 30 kVA. The four PESAs received the UHF waves successively, based on their locations. Furthermore, a four-channel digital oscilloscope (Tektronix, Beaverton, OR, USA, DPO7354C, 3.5 GHz, 40 GS/s) was employed to acquire and record the TPRD UHF patterns.

Five types of PDs were fabricated to simulate the various insulation defects, including the floating electrode, a metal protrusion on the conductor and the tank, surface contamination, and free metal particles. Besides, three relative angles between the defect position and the PESA antennas, which were 0°, 90°, and 180°, were integrated with the five defect types in the defect simulations.

**Figure 10:**
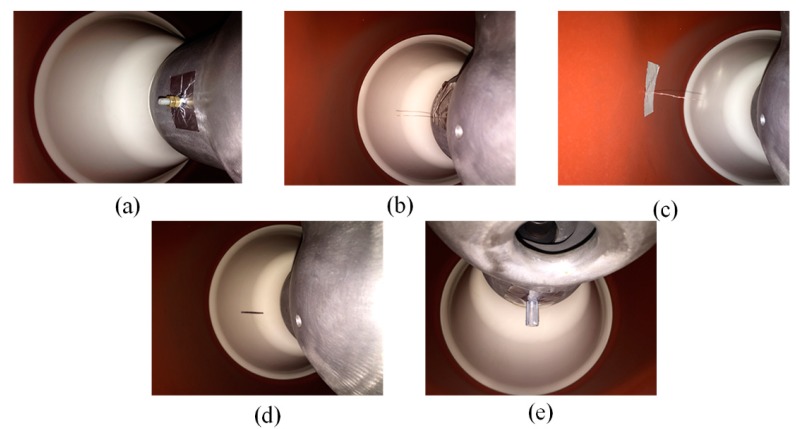
Typical PD defect patterns: (**a**) floating electrode; (**b**) metal protrusion on the conductor; (**c**) metal protrusion on the tank; (**d**) surface contamination; (**e**) free metal particles.

**Figure 11:**
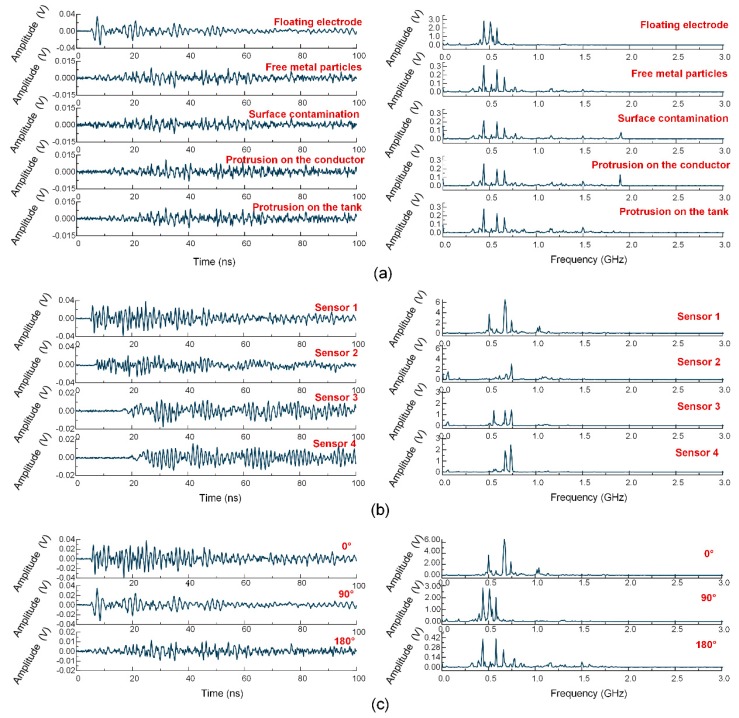
Typical UHF waveforms: (**a**) UHF signals of five different defect types; (**b**) UHF signals acquired from the four channels; (**c**) UHF signals of the three relative angles.

As shown in Figure 10, the floating electrode defect was simulated by fixing two adjacent copper nuts to an insulated bolt that was attached on the conductor. The nuts were 3 mm away from the conductor, and the distance between the copper nuts was 1 mm. Two kinds of metal protrusions were replicated by a 30 mm needle adhered on the high-voltage conductor and tank, respectively. Surface contamination was replicated by a piece of aluminum foil (2 × 20 mm^2^) adhered to the surface of the spacer. Finally, the free particles defect was produced by connecting an insulation tube that contained some aluminum particles inside the conductor, with the length of the insulation tube being 20 mm. The first four types of defects were arranged at three relative angles between the defect positions and the sensor position for 0°, 90°, and 180°, as shown in Figure 8, generating 13 PD conditions in total (the free metal particles were only simulated at 90°). Figure 11 shows some typical acquisitions of the UHF signals within 100 ns, and their corresponding frequency spectra.

## 5. Experiment Evaluations

A detailed analysis was carried out to ascertain the model’s discriminatory capability. Both the relative angles and defect types were taken into consideration, including the floating electrode (0°, 90°, and 180°), the metal protrusion on the conductor (0°, 90°, and 180°), the metal protrusion on the tank (0°, 90°, and 180°), the surface contamination (0°, 90°, and 180°), and the free particles (90° only), thus creating 13 defect modes. There were 1386 samples in total, of which 20% were used for testing. Two cases were designed for recognition with the same dataset, namely, (1) the combined diagnosis of positions and defect types together, which had 13 classes in total, and (2) diagnosis of the defect types only, which has five classes only.

### 5.1. Implementation Details

**Figure 12:**
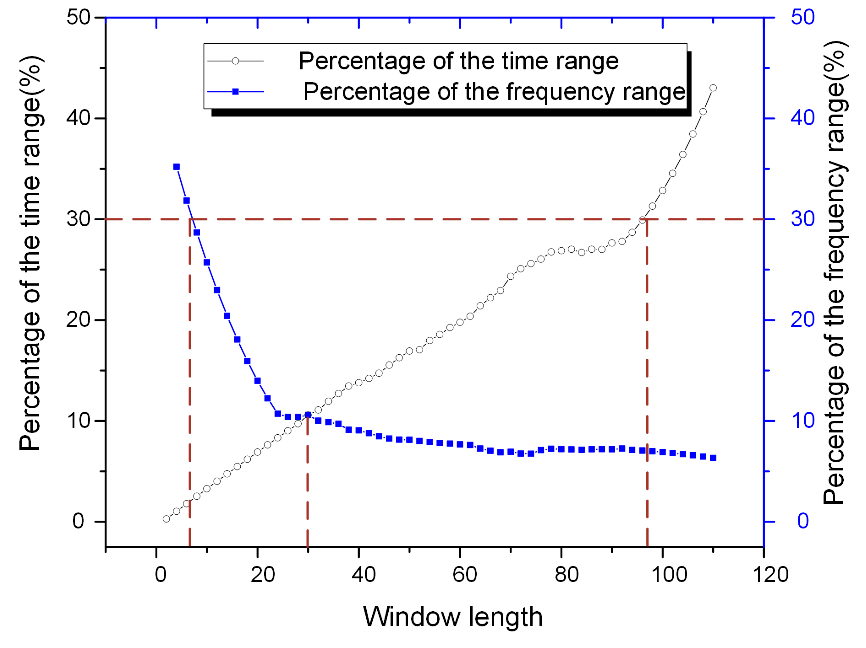
Percentages of the time and frequency decline ranges along the window lengths.

First, the lengths of the analysis windows were chosen based on the frequency decline range and the time decline range described in Figure 2, and 50% of the max value was chosen as the threshold. Instead of using the ranges directly, their proportions in the whole analysis time axis and frequency axis were calculated for ease of comparison. The time range limit and frequency range limit were chosen as 100 ns and 3 GHz, respectively. The sampling rate was 10 GHz, indicating that 0.1 ns is added when the window length increases by 1. Figure 12 shows the variations of the percentages of the average time and frequency ranges. It is obvious that as the window length increased, the frequency resolution increases as well, while the time resolution showed an opposite trend. In order to retain enough resolution information for both the axis, 30% was selected as the threshold. It is observed that the window lengths were near 6 and 100 at this point. Therefore, the temporal window and frequency window were chosen as 6 and 100, respectively. Besides, the crossing point was obtained near 30, which was selected as the window for the texture channel.

Besides, in order to further reduce the input dimension, a bigger stride was used along the time axis for the frequency channel, while a bigger frequency stride was utilized for the temporal channel in STFT, which could be seen as a subsampling. A cut-off of 2 GHz was also utilized in the temporal channel and the texture channel, considering that the details in the high-frequency band were trivial in their diagnosis. Finally, the windows and the input shapes of the three subnetworks are summarized in Table 2. Considering the limited amount of data, the neural network architecture was wide, but it had only a few convolutional maps per column. The detailed net structures are also shown in Table 2. The three columns are the time column, frequency column, and texture column, respectively.

The learning algorithm setup was as follows: the Adadelta optimizer with a learning rate of 1.0 for the pretraining and MLP embedding stages, and a Rmsprop optimizer for LSTM with a learning rate of 0.001 and epsilon of 1 × 10^−6^. The pretraining was carried out for 1000, 800, and 400 iterations, with the patience being 100, 50, and 50. All networks were realized by Keras and NVIDIA 750TI GPUs (NVIDIA, Santa Clara, CA, USA). The tests averaged over five sets of initialization parameters were reported as the final results.

### 5.2. PD Pattern Recognition Results

#### 5.2.1. Diagnosis Accuracies

The recognition accuracies of the two cases of (1) diagnosis of both the defect positions and types, and (2) the defect types only, are shown in Table 3 and Table 4. Overall, we achieved 97.51% testing accuracy for the recognition of both the angles and defect types, and 98.20% for the defect types only, based on the experimental dataset.

Furthermore, the diagnostic accuracies of single resolutions and single sensors with partial configurations are also presented as quantitative measures of the informativeness. The following points may be observed, based on the recognition accuracies.

1. Multi-resolution diagnosis leads to a smaller misclassification rate than by using only the single resolution information.
2. Sensor1 has the best accuracy among all the sensors with respect to the shortest distance to the defect location. The information loss can be quite significant after the L-shaped corner.
3. A higher frequency resolution is more valuable for single resolution diagnosis.
4. The multi-sensor combination is not certainly better than a single sensor without appropriate methods for the increasing the dimensionality and the confusing information carried by different sensors.

#### 5.2.2. Loss Curves and Time Consumption

**Figure 13:**
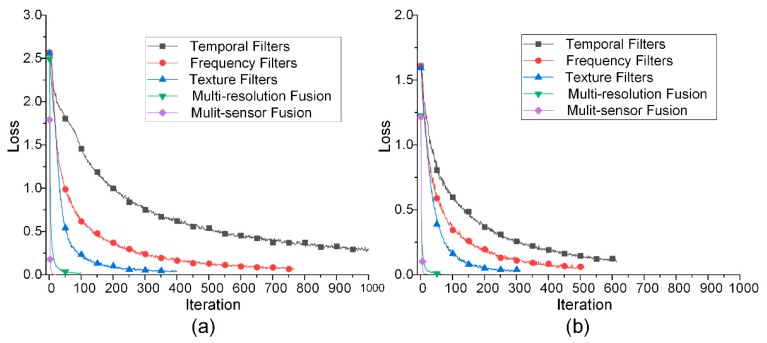
Loss curves of the three stages of the transfer learning: (**a**) The defect types and positions; (**b**) the defect types only.

**Figure 14:**
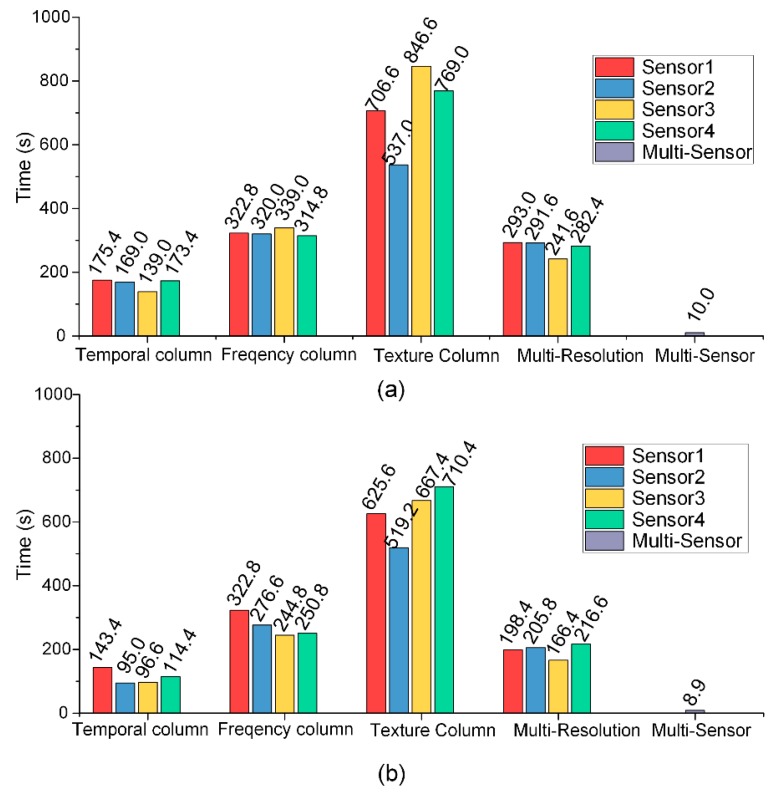
Time consumptions of the three stages of the transfer learning: (**a**) The defect types and positions; (**b**) the defect types only.

To diagnose the effectiveness of transfer learning, the loss curves and time consumptions of the single column training, multi-column training, and the LSTM training are shown in Figure 13 and Figure 14. It is obvious that during the pretraining stage, the convergence speed of the texture channel was the fastest, while the frequency channel ranked second, and the temporal channel was the slowest. However, the time consumption was the other way around. The texture channel called for the longest training time. This was attributed to the number of dot calculations for each iteration being much more than the other two kinds of kernels. Besides, both the iteration number and the time consumption of the second and third training stages were much less, compared with their previous steps. For the final LSTM information fusion stage, only less than 10 s was needed.

The results in Table 3 and Table 4, and Figure 13 and Figure 14 show the exhaustive role played by transfer learning. It is significant that during the three training stages, the diagnostic accuracy increased, while both the training iterations and time decreased dramatically, even with a larger network. This dynamic indicates that the knowledge learned from earlier stages was successfully transferred to a higher level with less proneness to overfitting.

#### 5.2.3. Visualization of the Convolutional Filters

**Figure 15:**
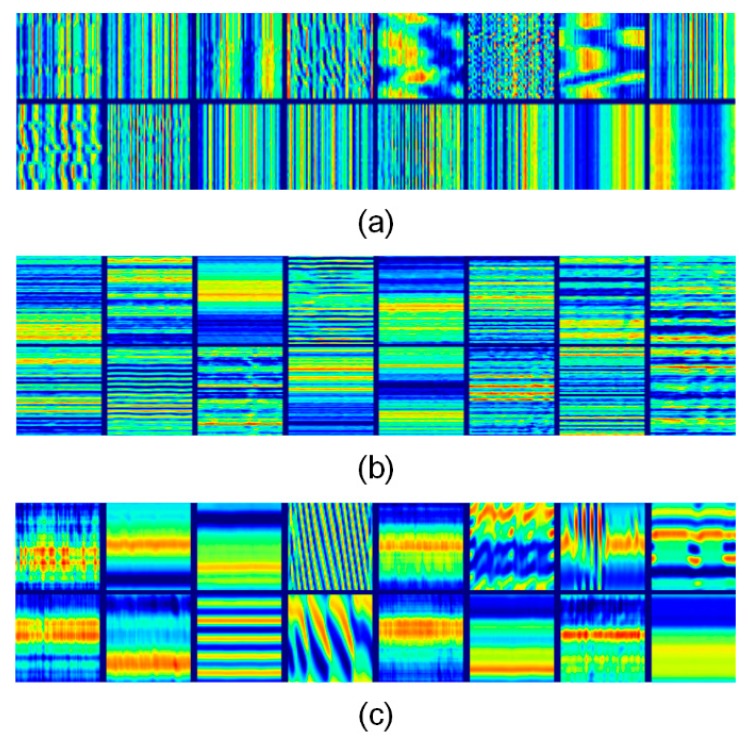
Visualization of the convolutional filters: (**a**) temporal filters; (**b**) frequency filters; (**c**) texture filters.

Neural networks are usually considered as black boxes. However, with the help of visualization methods, it is possible to obtain good qualitative interpretations of the black boxes of deep networks. Besides, visualization is also an effective way to diagnose the misclassifications and evaluate the degree of overfitting. In this study, the Activation Maximization [49] method was utilized to visualize the learned filters. The idea of Activation Maximization is quite simple: generating an image to maximize the activation of a hidden unit as:

(17) $$\;x^{*}={\mathrm{argmax}}_{x\;s.t.\;\|x\|=\rho }h_{ij}\left(\theta ,\;x\right)\;$$

where $x^{*}$ is the visualization result, and θ donates the fixed neural network parameters. Thus, $h_{ij}\left(\theta ,\;x\right)$ is the activation values of unit j in layer i with fixed parameters θ and bounded norm ρ. For simplicity and the convenience of comparison, only the first layer of filters were visualized, as shown in Figure 15.

It is clear that the learned convolutional filters were quite smooth in space, thus indicating that the training was sufficient. The temporal filters were sensitive to pulses along the time axis, while the frequency filters showed clear patterns of different frequency combinations. Besides, the diversity of patterns was richer in the visualization results of the texture filters, where both the temporal and frequency patterns revealed their importance. It is also encouraging that both the temporal filters and frequency filters gained significant resolution power over multiple scales, as shown by the different densities of the lines.

**Figure 16:**
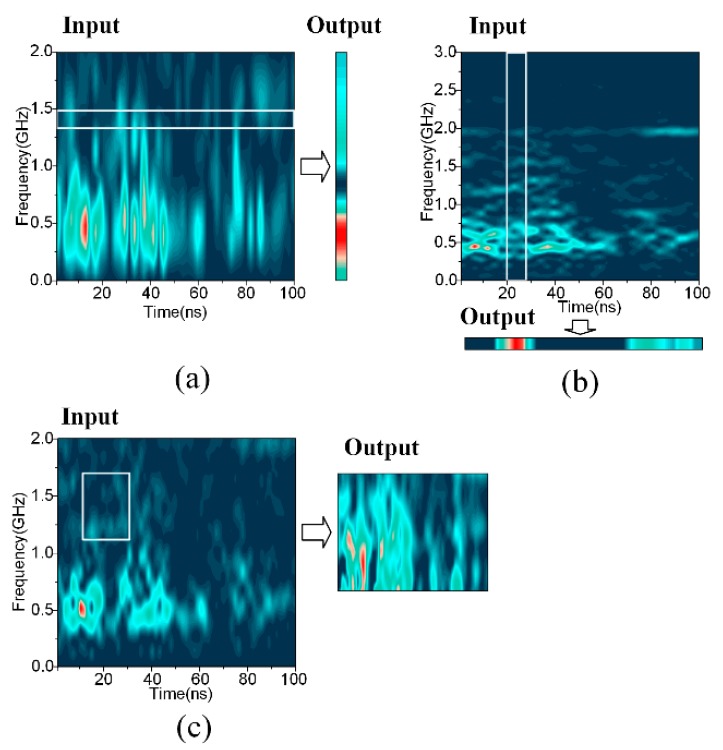
The outputs of the first layer of convolutional filters. (**a**) The output of the temporal filter; (**b**) the output of the frequency filter; (**c**) the output of the texture filter.

Besides, a direct visualization of the outputs of the filters was helpful in illustrating the automatic feature extraction and the information compression of the filters. An illustrative example is shown in Figure 16. The outputs of the frequency filter and the temporal filter were only one-dimensional, while the texture filter transferred the two-dimensional input to a smaller feature map. That is also why the texture filter calls for the longest training time.

#### 5.2.4. Visualization of the Learned Embedding Features

**Figure 17:**
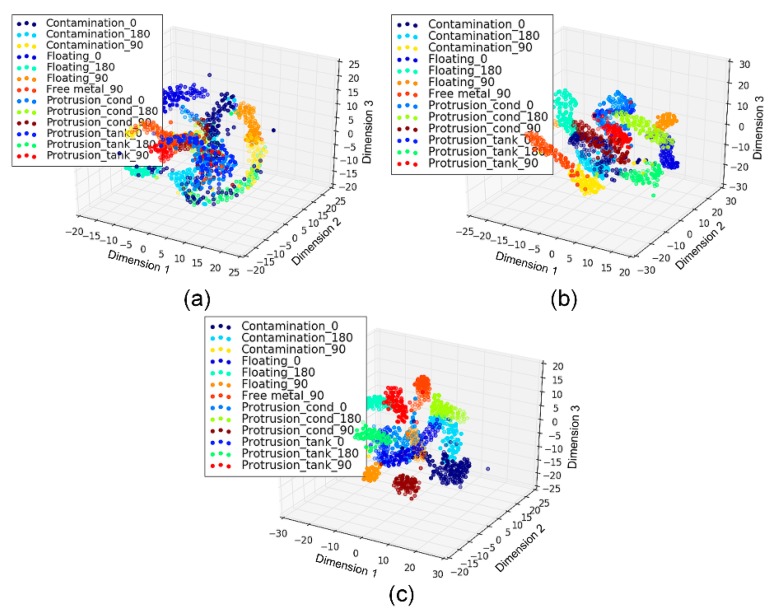
Visualization of the learned features for both the defects and positions: (**a**) Temporal channel; (**b**) frequency channel; (**c**) texture channel.

**Figure 18:**
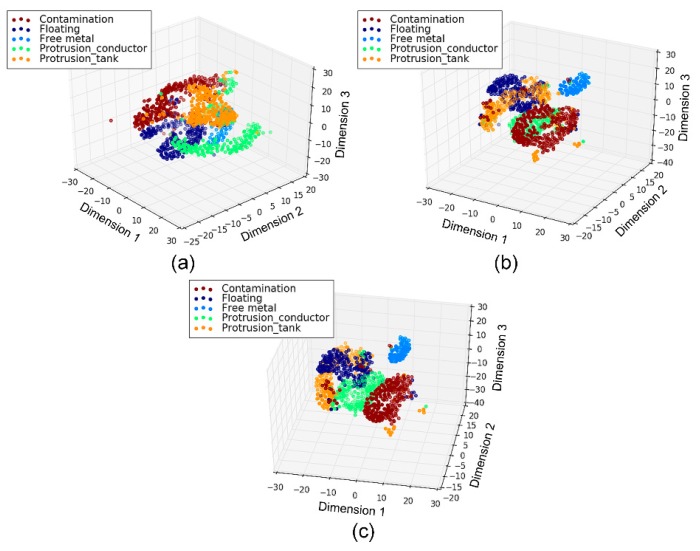
Visualization of the learned features for the defect types only: (**a**) Temporal channel; (**b**) frequency channel; (**c**) texture channel.

Furthermore, by using a t-Distributed Stochastic Neighbor Embedding (t-SNE) projection algorithm, the 50-dimensional features learned by the subnetworks were reduced to three dimensions, as shown in Figure 17 and Figure 18 for the cases of 13 and five classes, respectively. It was observed that the features learned automatically by the CNN of different defects fell into distinct clusters in the three-dimensional space, where each cluster showed clear manifold characteristics of a continuous distribution in the space.

### 5.3. Comparison with the Baselines

To further verify the effectiveness, some baselines were implemented. Instead of focusing on the specified methods, we first extracted the common framework utilized in the practice of UHF signal recognition, which were two-dimensionalization, matrix compression, decomposition, features extraction, feature selection (FS), and the final classifier. Second, by using typical models in certain steps, representative methods could be obtained. A description of some implemented techniques is as follows.

1. Two-dimensionalization: Wavelet spectrum and Hibert–Huang spectrum;
2. Matrix compression: NMF and 2DPCA;
3. Signal Decomposition: Wavelet transform and Hibert–Huang transform (HHT);
4. Features extraction: The extracted time and frequency (T&F) features include the max value, root mean square deviation, standard deviation, skewness, kurtosis, and the peak-to-peak value in the time domain. The frequency features include the mean frequency, frequency center, root mean square frequency, and the standard deviation frequency. Besides, the entropy is also calculated.
5. Feature selection: ExtraTrees Classifier and LinearSVC feature selection;
6. Classifier: Finally, both the SVM and DNN of dense layers are chosen as the final classifiers, due to their high representativeness in engineering practice.

Through the combination of the different techniques, 18 diagnosis models are summarized in Table 5 as the baselines, including both simple methods and complicated flows. However, the recognition accuracies are not certainly proportional to the complexity of the methods.

All of the hyperparameters are selected based on cross-validation to the best of our knowledge. The final recognition accuracies of (1) combined the diagnosis of positions and defect types together, 13 classes, and (2) diagnosis of the defect types only; five classes are shown in Table 6 and Table 7, respectively.

The accuracy comparisons may provide some insights into the fundamental differences between the traditional feature-based methods and the deep learning approaches, as well as some essential flaws of the feature engineering methods.

1. The misclassification rate of the proposed multi-resolution CNN clearly indicates its superior capability compared with the baseline methods. STFT’s performance is slightly better than the Wavelet spectrum in certain resolutions, and much better than the HHT map.
2. The best performance in the baseline methods is gained by the combination of STFT, NMF, and SVM, which is 92.09% for the 13 classes, and 93.88% for the five classes. Firstly, it is observed that NMF slightly outperforms 2DPCA, which may be explained as follows. In the experiments, the NMF gains the best performance with four dimensions, compared with the 12 dimensions of the 2DPCA, indicating that the redundancy is huge in the STFT maps. Thus, searching for a global projection in the redundancy data may be not a good idea. Second, the features extraction usually does not increase the model performance, which reflects the eternal contradiction among the learning ability of the model, the input dimensions, the distribution of the data, and the discernibility of the features.
3. The performances of SVM and DNN are similar when utilizing the same input features. Therefore, for traditional feature-based recognition methods, the recognition accuracy relies much on the discriminative features.
4. Similar to the conclusion drawn from Table 3 and Table 4, the performance of multiple sensors is not certainly better than the single sensor, especially when the nearest single sensor can gain good diagnostic accuracy.

## 6. Discussion

Although the deep networks have gained tremendous performance in many areas, low interpretability is always an obstacle to applying them in some highly regulated environments, due to the uncontrollability that comes with the unclear mechanism. Sometimes, the training of the neural networks becomes a trial-and-error problem. In recent years, a great number of studies have focused on the interpretability of deep learning, and great progress has been made in both the theories and experiments. In the discussion, instead of pursuing a general solution, we only concentrate attention on the special CNN structures that are implemented here, and try to explain its effectiveness from the perspective of manifold learning.

**Figure 19:**
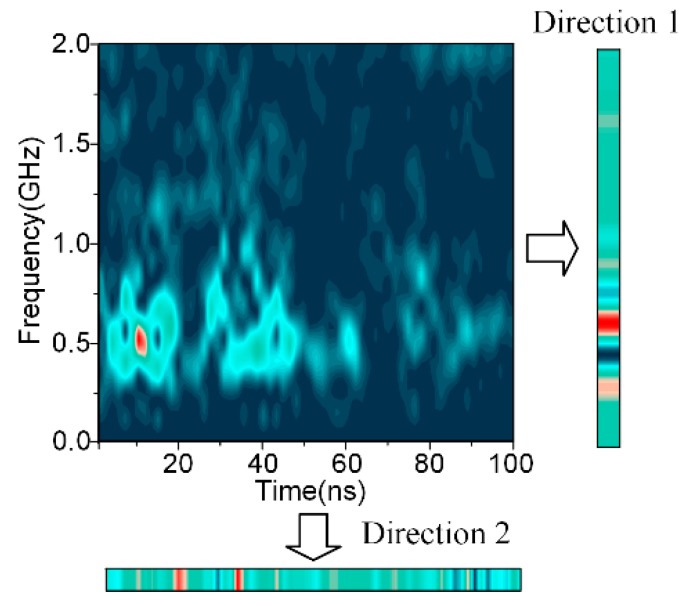
Visualization of the compressed results of the 2DPCA.

A comprehensive comparison of the similarities between the filters learned by the CNN and the matrix projection-based methods, such as 2DPCA, is presented to provide some insights into the advantages of deep learning methods, compared with the feature extraction solutions. It is quite interesting to notice the similarity between the projection operation in 2DPCA and the convolutions in CNN. In 2DPCA, an image A of m×n matrix is projected into a lower dimension by:

(18) $$Y_{k}=AX_{k}$$

where $X_{k}$ is a vector of n dimensions, and k is the index. A visualization example of the 2DPCA’s compressed result of the STFT spectrum is shown in Figure 19. It is easy to notice its similarity with the outputs in Figure 16, while the information concentration in Figure 16 is better.

Moreover, it is also possible to reconstruct the original image from the compressed information by:

(19) $$\hat{A}=\overset{d}{\underset{k=1}{\sum }}Y_{k}X_{k}^{T}$$

where d is the compression dimension. Therefore, in 2DPCA, it is assumed that any input can be approximately reconstructed as a weighted sum of smaller collections. In summary, the key points in 2DPCA include:

(1) The projection operation for compressing,
(2) The assumption that complex images are composed of eigenimages.

It is quite interesting that similar characteristics are shared by the proposed temporal and frequency filters.

(1) The dot production between the frequency filters and the spectrum, and the similar operation between the temporal filters and the spectrum can be seen as a projection operation,
(2) In the CNN, it is also assumed that images are formed from elements of the lower level, like pixels to more composite representations [28].

Therefore, a common framework that distinguishes different categories by matrix projection may be extracted to unify the deep structure and 2DPCA. The CNN and 2DPCA both try to classify different categories by using matrix projections, and the main differences lies in their supervision methods, where the CNN is supervised by labels, while 2DPCA is supervised by the inner product in a high-dimensional space. Furthermore, a special case of NMF that aims at reducing the reconstruction errors by projecting could also be defined to fit this framework. The main specialty lies in that the loss function is defined at the single sample, and it uses the mean square error between the original images and the reconstructed ones.

Moreover, based on this framework, the 2DPCA can also be reformulated and approximated as a special case of the CNN with the following setups: (1) Use only a single layer of convolutions, and the width of the convolutional kernels should be restricted at one. (2) The loss function is defined as the sum of the pairwise Euclidean distance losses to force the model to maintain its relative relations in higher space. Some new algorithms may be developed based on this idea.

Therefore, the superiority of the proposed may be explained as follows. Based on the same framework, only the CNN is goal-oriented at the final classifying task, while the 2DPCA and NMF aim at the other specified goals. It is usually hard to determine the effectiveness of these specified goals until obtaining the final results. Is it better to maintain the global information as 2DPCA, or to keep the local completeness as in NMF? In addition, the multiple projections also equip the CNN with a better learning ability to handle more complex problems.

## 7. Conclusions

Inspired by the phenomenon that the PD spectrograms of different resolutions can capture the information of different patterns, both multi-resolution and multi-sensor fusion algorithms are proposed. They are realized by a multi-column deep CNN characterized by specified filters and a sequential LSTM network. A multi-stage transfer learning framework is also presented to train the model with limited data. Several conclusions may be drawn based on the detailed analysis.

1. The proposed multi-resolution model gains accuracies of 97.51% and 98.20% on the tasks of diagnosing (1) the positions and defect types together, and (2) the defect types only; thus indicating its clear superiority compared with the baselines.
2. The loss curves, time consumptions, and diagnostic accuracies show the effectiveness of transfer learning, which successfully transfers knowledge from a lower level to a higher level with less proneness to overfitting.
3. The comparisons with the baseline methods reveal several fundamental flaws of the feature based methods, such as the difficulties in choosing the most suitable approach, and the curse of dimensionality when encountering too many features from multiple sensors.
4. A matrix projection framework is proposed to enhance the interpretability of the proposed deep network structure.

## Figures and Tables

**Table 1:** Summary of the relevant features and classifiers that have been used for partial discharge (PD) recognition.

Reference	PD Types	Feature Extraction	Classifier	Recognition Accuracy
Umamaheswari and Sarathi [9]	Four types of artificial insulation defects	Partial power analysis	SVM	Average of 95.25%
Darabad et al. [10]	Ten types of PD sources	Texture features + PCA	SOM	Grouped data visualization
Mas’ud et al. [11]	Six PD fault geometries in oil, air, and poly-ethylene-terephthalate	Statistical parameters from the PRPD patterns	ENN	Average 95%
Evagorou et al. [12]	Four types of artificial PDs in oil and air.	Wavelet packet transform + statistical parameters	PNN	97.49%, 91.9%, 100%, and 99.8%
Wang et al. [13]	Four types of artificial defect models in the oil and air.	S-transform	Affinity propagation clustering	99.67%
Li et al. [14]	Four kinds of typical defects in a 252 kV GIS	Cumulative energy in time and frequency domain + mathematical morphological gradient	Fuzzy maximum-likelihood algorithm	98%
Li et al. [15]	Four kinds of defects in GIS	Statistical parameters of both the TRPD and PRPD patterns	Dempster–Shafer evidence theory + Neural network	97.25%
Albarracín et al. [16]	Separation of PDs with electromagnetic noises	Power ratios and the signal times	Grouped data visualization	The separation criteria was given
Li et al. [17]	Six types of artificial insulation defect models in the oil, air, and paper-fiber interface.	Wavelet packages + fractal features + Linear discriminant analysis	Finding the closest centroid	99.4, 94.5, 99.4, 91.9, 87.5, and 97.7%, respectively.
Wang et al. [18]	Three typical PD defect patterns in a 252 kV GIS	Fourier transform + Chromatic methodology	SVM	86.67%
Gu et al. [19]	Three common defect types of 25 kV cross-linked polyethylene (XLPE) power cable	HHT + Fractal parameters	NN	100%, when 5% random white noise
Dai et al. [20]	Four kinds of artificial defects in GIS	S-transform + Singular value decomposition (SVD)	SVM	98.33%
Majidi et al. [21]	Air voids with dimensions of 1, 1.5, and 2 mm	1-norm, 2-norm, and infinity-norm of the statistical features	Sparse representation vector	99.7%, 92.9%, 94.0%, and 81.6% for four different scenarios
Khan et al. [22]	Parallel-plane stainless steel electrodes in SF6 with 10 different particle lengths and positions	Statistical features + PCA	NN	88%

**Table 2:** Detailed network structure.

Index	Window	Input Shape	CNN Structures	MLP	LSTM
Column 1	6	30 × 50	(Conv2 × 50−MaxPooling2 × 1−Dropout)−(Conv2 × 1−MaxPooling2 × 1−Dropout)−(Dense100−Dense50)	Flatten–Concatenation–Dense100–Dense50	LSTM with 16 inner nodes–Flatten–Dense50–Output
Column 2	100	150 × 36	(Conv150 × 4−MaxPooling1 × 2−Dropout)–(Conv1 × 3–MaxPooling1 × 2−Dropout)–(Dense100−Dense50)		
Column 3	30	100 × 64	(Conv50 × 4−MaxPooling2 × 2−Dropout)−(Conv2 × 2−MaxPooling2 × 2−Dropout)−(Dense100−Dense50)		

**Table 3:** Diagnostic accuracies and the intermediate outputs for classifying both the positions and defect types.

Index	Structure	Single Sensor Diagnosis (%)				Multi-Sensor (%)
		Sensor1	Sensor2	Sensor3	Sensor4	
1	Temporal column	85.72	86.94	66.62	71.94	87.91
2	Frequency column	94.57	93.56	85.43	83.78	95.35
3	Texture column	94.42	93.05	84.78	86.30	93.70
4	Multi-resolution	96.08	95.07	91.01	90.18	97.51

**Table 4:** Diagnostic accuracies and the intermediate outputs for classifying the defect types only.

Index	Structure	Single sensor Diagnosis (%)				Multi-Sensor (%)
		Sensor1	Sensor2	Sensor3	Sensor4	
1	Temporal column	89.53	90.86	81.22	81.04	94.53
2	Frequency column	95.58	96.29	90.14	87.81	96.51
3	Texture column	96.15	95.07	89.53	87.48	95.79
4	Multi-resolution	97.37	97.09	91.29	90.79	98.20

**Table 5:** Typical diagnosis models for comparisons.

Index	Summary	Two-Dimensionalization Analysis		Feature Extraction		Feature Selection	Classifier
		Two-Dimensionalization	Matrix Compression	Decomposition	Features		
1	Map + CNN	HHT Map	-	-	-	-	CNN
2		Wavelet Map	-	-	-	-	CNN
3	Raw input	-	-	-	-	Yes	SVM
4		-	-	-	-	Yes	DNN
5	T&F features	-	-	-	T&F	Yes	SVM
6		-	-	-	T&F	Yes	DNN
7	Wavelet + T&F	-	-	wavelet	T&F	Yes	SVM
8		-	-	wavelet	T&F	Yes	DNN
9	HHT + T&F	-	-	HHT	T&F	Yes	SVM
10		-	-	HHT	T&F	Yes	DNN
11	STFT + NMF	STFT	NMF		-	Yes	SVM
12		STFT	NMF		-	Yes	DNN
13	SFTF + NMF + T&F	STFT	NMF		T&F	Yes	SVM
14		STFT	NMF		T&F	Yes	DNN
15	SFTF + 2DPCA	STFT	2DPCA			Yes	SVM
16		STFT	2DPCA			Yes	DNN
17	SFTF + 2DPCA + T&F	STFT	2DPCA		T&F	Yes	SVM
18		STFT	2DPCA		T&F	Yes	DNN

**Table 6:** Diagnostic accuracies of the baseline methods for classifying both defect types and positions.

Index	Summary	Single Sensor (%)				Multi-Sensor (%)
		Sensor1	Sensor2	Sensor3	Sensor4	
1	HHT spectrum + CNN	65.58	61.73	45.94	48.49	60.79
2	Wavelet spectrum + CNN	90.72	91.83	84.82	84.46	90.61
3	FS + SVM	89.20	87.41	67.27	66.18	66.18
4	FS + DNN	89.17	86.58	67.66	62.99	78.77
5	T&F + FS + SVM	55.04	55.04	41.73	49.64	72.30
6	T&F + FS + DNN	55.58	56.73	43.20	46.37	74.31
7	Wavelet + T&F + FS + SVM	61.87	55.76	47.48	49.64	76.61
8	Wavelet + T&F + FS + DNN	65.07	58.02	46.62	50.58	76.87
9	HHT + T&F + FS + SVM	37.33	32.73	22.66	32.01	45.68
10	HHT + T&F + FS + DNN	38.41	36.76	30.14	33.45	42.58
11	STFT + NMF + FS + SVM	90.29	88.85	72.66	68.70	92.09
12	STFT + NMF + FS + DNN	90.93	90.58	76.12	71.04	91.55
13	STFT + NMF + T&F + FS + SVM	77.70	71.58	56.83	57.91	85.97
14	STFT + NMF + T&F + FS + DNN	80.14	72.91	59.57	58.09	84.86
15	STFT + 2DPCA + FS + SVM	87.41	84.53	57.19	55.39	87.05
16	STFT + 2DPCA + FS + DNN	87.62	84.10	60.28	55.36	85.89
17	STFT + 2DPCA + T&F + FS + SVM	84.53	76.25	49.28	49.64	82.37
18	STFT + 2DPCA + T&F + FS + DNN	84.06	76.47	54.60	49.78	81.72

**Table 7:** Diagnostic accuracies of the baseline methods for classifying the defect types only.

Index	Summary	Single Sensor (%)				Multi-Sensor (%)
		Sensor1	Sensor2	Sensor3	Sensor4	
1	HHT Spectrum + CNN	73.02	74.1	60.68	60.86	76.47
2	Wavelet Spectrum + CNN	92.41	93.81	90.32	89.82	91.94
3	FS + SVM	92.45	90.29	73.37	71.94	81.29
4	FS + DNN	90.72	89.28	76.80	76.16	77.48
5	T&F + FS + SVM	64.39	60.07	52.52	62.95	81.29
6	T&F + FS + DNN	67.63	68.71	56.87	66.22	82.01
7	Wavelet + T&F + FS + SVM	68.35	66.91	57.55	61.51	82.73
8	Wavelet + T&F + FS + DNN	72.60	67.63	60.07	65.00	81.85
9	HHT + T&F + FS + SVM	48.92	50.00	42.81	48.56	56.83
10	HHT + T&F + FS + DNN	54.93	54.14	44.82	53.53	60.07
11	STFT + NMF + FS + SVM	91.01	90.65	82.37	77.70	93.88
12	STFT + NMF + FS + DNN	92.59	90.72	83.67	76.73	93.48
13	STFT + NMF + T&F + FS + SVM	79.50	74.82	68.35	69.78	88.49
14	STFT + NMF + T&F + FS + DNN	84.03	78.13	70.36	68.71	89.28
15	STFT + 2DPCA + FS + SVM	87.76	87.76	66.19	67.63	87.41
16	STFT + 2DPCA + FS + DNN	89.71	86.01	70.14	71.94	86.47
17	STFT + 2DPCA + T&F + FS + SVM	86.69	80.58	64.03	63.31	87.05
18	STFT + 2DPCA + T&F + FS + DNN	85.79	82.41	61.83	65.32	84.38
